# Hsp70 in Redox Homeostasis

**DOI:** 10.3390/cells11050829

**Published:** 2022-02-28

**Authors:** Hong Zhang, Weibin Gong, Si Wu, Sarah Perrett

**Affiliations:** 1 National Laboratory of Biomacromolecules, CAS Center for Excellence in Biomacromolecules, Institute of Biophysics, Chinese Academy of Sciences, 15 Datun Road, Chaoyang District, Beijing 100101, China; gongweibin@ibp.ac.cn (W.G.); wusi@ibp.ac.cn (S.W.); 2 University of the Chinese Academy of Sciences, 19 Yuquan Road, Shijingshan District, Beijing 100049, China

**Keywords:** redox homeostasis, oxidative stress, ROS, Hsp70, cysteine modifications, glutathionylation

## Abstract

Cellular redox homeostasis is precisely balanced by generation and elimination of reactive oxygen species (ROS). ROS are not only capable of causing oxidation of proteins, lipids and DNA to damage cells but can also act as signaling molecules to modulate transcription factors and epigenetic pathways that determine cell survival and death. Hsp70 proteins are central hubs for proteostasis and are important factors to ameliorate damage from different kinds of stress including oxidative stress. Hsp70 members often participate in different cellular signaling pathways via their clients and cochaperones. ROS can directly cause oxidative cysteine modifications of Hsp70 members to alter their structure and chaperone activity, resulting in changes in the interactions between Hsp70 and their clients or cochaperones, which can then transfer redox signals to Hsp70-related signaling pathways. On the other hand, ROS also activate some redox-related signaling pathways to indirectly modulate Hsp70 activity and expression. Post-translational modifications including phosphorylation together with elevated Hsp70 expression can expand the capacity of Hsp70 to deal with ROS-damaged proteins and support antioxidant enzymes. Knowledge about the response and role of Hsp70 in redox homeostasis will facilitate our understanding of the cellular knock-on effects of inhibitors targeting Hsp70 and the mechanisms of redox-related diseases and aging.

## 1. Introduction

Cellular redox homeostasis relies on precise coordination between the generation and elimination of reactive oxygen species (ROS). ROS can act as signaling molecules to modulate transcription factors and epigenetic pathways controlling cell survival and death [1,2]. Cellular ROS levels undergo continuous fluctuations within the physiological redox range. The redox status of the cell is essential to regulate basic biological processes including cell proliferation/differentiation, metabolic homeostasis and immune responses [2,3]. Oxidative stress occurs when ROS generation exceeds ROS clearance and the redox status is outside the physiological redox range [4]. Continuous oxidative stress leads to cell, tissue and organ damage and is linked to pathogenesis in cancer, diabetes, cardiovascular disease, neurodegenerative diseases, reproductive system diseases and the process of aging [5].

ROS types can be divided into free radicals and nonradicals. The free radicals include nitric oxide (NO^•^), the superoxide radical anion (O_2_^•−^), the hydroxyl radical (OH^•^), the carbonate radical anion (CO_3_^•−^), nitrogen dioxide (NO_2_^•^) and alkoxyl/alkyl peroxyl (RO^•^/ROO^•^). Hydrogen peroxide (H_2_O_2_), peroxynitrite (ONOO^−^)/peroxynitrous acid (ONOOH) and hypochlorous acid (HOCl) are the major nonradicals. ROS generation includes exogenous input and endogenous production from the mitochondrial electric transport chain, as well as the catalytic products of the enzymes nitric oxide synthase (NOS), NADPH oxidase (NOX) and myeloperoxidase. ROS can be transformed from one type to another by a sequence of reactions. The clearance of ROS relies on both enzymatic and nonenzymatic antioxidants. Enzymatic antioxidants include superoxide dismutase (SOD), catalase (CAT), the glutathione peroxidase (GPX) system, the thioredoxin (TRX) system and peroxiredoxin (PRDX). Nonenzymatic antioxidants include glutathione (GSH), uric acid, Vitamin E, Vitamin C and bilirubin [3].

Oxidative stress can cause oxidation of proteins, lipids and DNA, potentially leading to cytotoxicity and/or cell death, but oxidative stress can also result in modification of protein cysteine residues, allowing the transfer of redox signals within different cellular signal transduction pathways [6,7,8,9,10]. Protein thiols can act as nucleophiles to undergo one- and two-electron oxidation reactions, forming thiol radicals or sulfenic acids for further reaction [11]. Reactions between ROS and protein thiols leads to multiple post-translational modifications (PTMs), including S-sulfenation (−SOH), S-sulfination (−SO_2_H), S-sulfonation (−SO_3_H), S-nitrosylation (−SNO), persulfidation (S-sulfhydration, −SSH), S-glutathionylation (−SSG) and disulfide bond formation (−S–S−) [12]. Modification of critical signaling molecules driven by ROS initiates signaling in a broad range of cellular processes [5,9,10,13] (Table 1).

**Figure 1:**
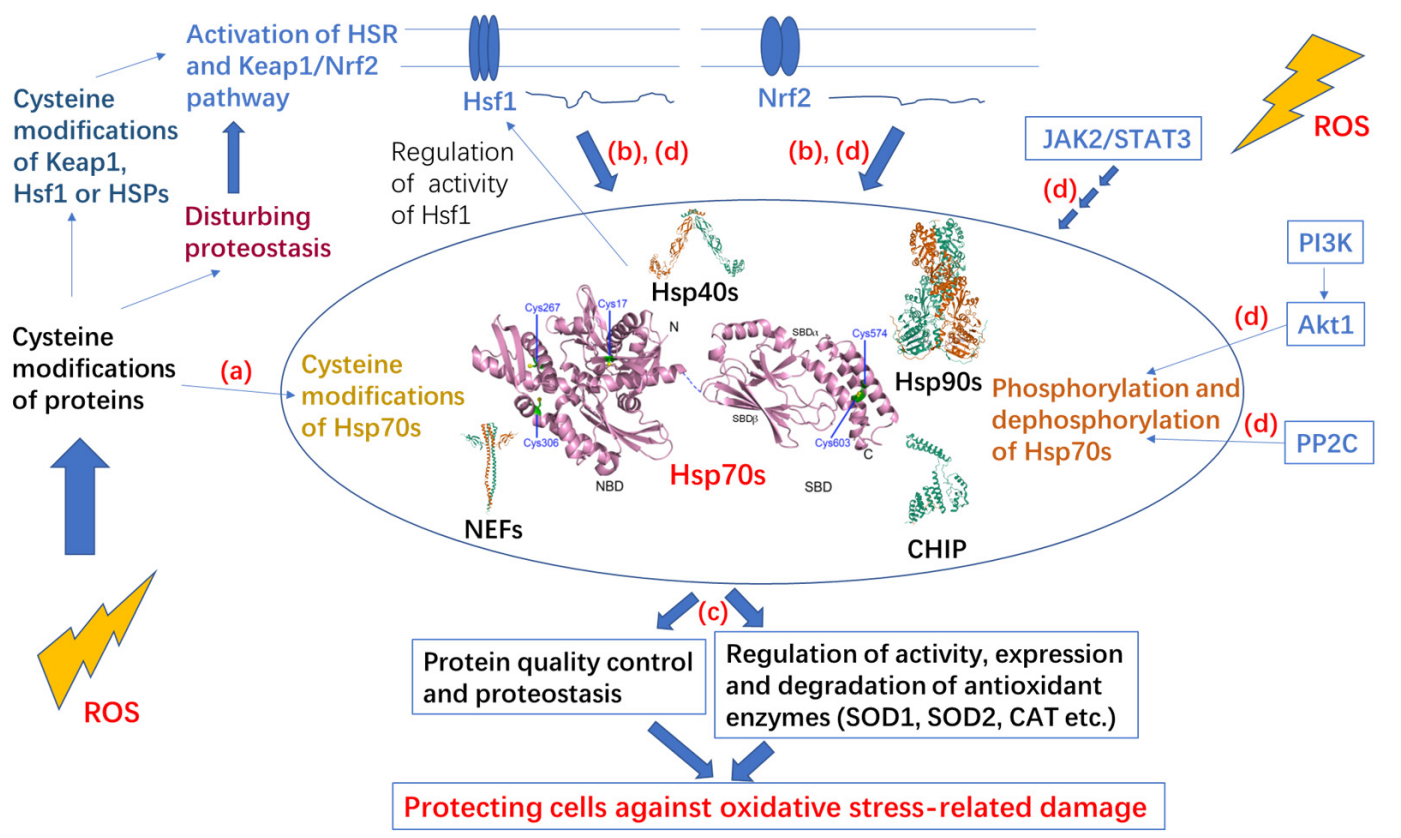
Scheme of Hsp70 in redox homeostasis. Reactive oxygen species (ROS) often cause extensive cysteine modifications of proteins and disturb proteostasis, which can activate the heat shock response (HSR) and Kelch-like ECH-associated protein 1 (Keap1)/nuclear factor erythroid 2-related factor 2 (Nrf2) pathways by activation of heat shock transcription factor 1 (Hsf1) and redox-sensitive transcription factor Nrf2. ROS also widely modify different cellular signaling pathways. Hsp70s are involved in redox homeostasis in several aspects: (**a**) ROS can cause cysteine modifications of Hsp70s to modify their functions; (**b**) activation of Hsf1 and Nrf2 elevate expression of *HSP70*s; (**c**) Hsp70s work as central hubs in the protein quality control network to maintain proteostasis, including eliminating oxidized proteins and regulating activity, expression and degradation of antioxidant enzymes to contribute to redox homeostasis; and (**d**) Hsp70s are involved in redox-related signaling pathways, leading to phosphorylation or upregulated expression of Hsp70s.

Oxidative stress can also activate heat shock transcription factors (HSFs) and redox-sensitive transcription factor nuclear factor erythroid 2-related factor 2 (Nrf2) by multiple mechanisms to induce gene expression related to cytoprotection including heat shock proteins (HSPs) and antioxidant enzymes [14,15,16]. Oxidation of proteins by ROS often results in damage to protein structure and alteration of protein function, thus disturbing protein homeostasis (proteostasis) [17]. Hsp70 is the key chaperone in protein quality control and the central hub of the cellular proteostasis network, participating in numerous cellular processes by interacting with different clients [18]. Hsp70 is tightly related to redox homeostasis in several ways, including functional regulation of Hsp70 caused by PTMs (especially cysteine modifications), induced expression of Hsp70 caused by oxidative stress, Hsp70-dependent proteostasis under oxidative stress and redox-related signaling pathways involving Hsp70 (Figure 1).

## 2. Hsp70 System

Hsp70 members are highly conserved, ubiquitous and extremely important for maintenance of life in all organisms apart from some archaea [19,20]. Hsp70 members are multifunctional proteins and participate in multiple processes related to protein quality control, including de novo protein folding, refolding of misfolded proteins, protein transport, assembly and disassembly of protein complexes, prevention of protein aggregation, solubilizing aggregated proteins, translocation of proteins across membranes, regulating protein activity, cooperating with downstream chaperones such as Hsp90, which is responsible for the final folding and maturation of some substrate proteins, and cooperating with the cellular degradation machinery [21]. Hsp70 members not only have housekeeping functions to promote maturation of clients and adjust their activity in different cellular processes but also guard cells against disruption of protein homeostasis due to proteotoxic stresses, pathophysiological conditions and organismal aging [21,22,23]. From bacteria to humans, there are multiple Hsp70 members that exert overlapped and nonoverlapped functions, and more complicated regulation mechanisms of Hsp70 have evolved to adapt to the complex clients and cellular environment in higher organisms [24,25]. There are three Hsp70 members in *Escherichia coli*, 14 members in *Saccharomyces cerevisiae* and 17 members in *Homo sapiens* (including several Hsp110 proteins which share a similar structure with typical Hsp70s and generally act as nuclear exchange factors (NEFs) of typical Hsp70s) (Table 2). In eukaryotes, different Hsp70 members are often expressed in distinct cellular compartments (e.g., cytosol, nucleus, ER or mitochondria), and the expression levels are regulated as required under different conditions, such as growth, stress, disease, aging and tissue-specific activities (Table 2) [25].

**Figure 2:**
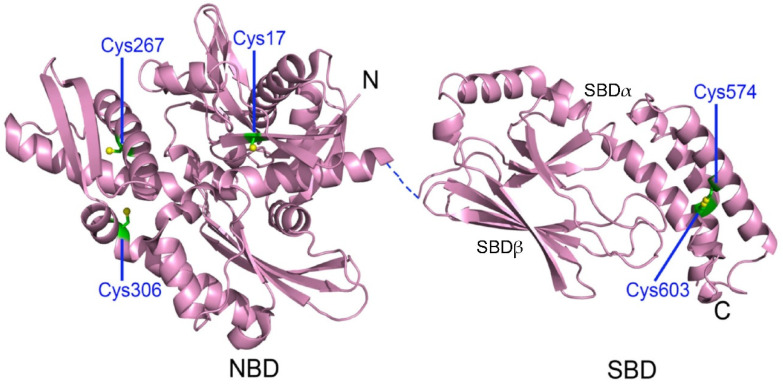
Crystal structures of human HspA1A. The nucleotide-binding domain (NBD) in the ADP-bound state (PDB code 3AY9) and the substrate-binding domain (SBD, PDB code 4PO2) are shown. The dashed line represents the flexible linker between the NBD and SBD. The SBD contains a β-sandwich substrate-binding subdomain (SBDβ) and an α-helical lid subdomain (SBDα). The five Cys residues are labeled in green. Figure reproduced from ref. [52].

Hsp70 members often share conserved sequence and structure, and the canonical Hsp70 structure is composed of an N-terminal nucleotide-binding domain (NBD) and a C-terminal substrate-binding domain (SBD) joined by a flexible linker (Figure 2) [26]. The NBD has an actin-like fold containing two lobes (I and II), which can be further subdivided into four subdomains (IA, IB, IIA and IIB) (Figure 2) [27]. The deep cleft between the two lobes accommodates binding of ATP/ADP with the interaction with all four subdomains, controlling lobe movements [28]. The SBD consists of a β-sandwich substrate-binding subdomain (SBDβ) containing around eight β-strands and linking loops, an α-helical lid subdomain (SBDα) containing four to five α-helixes and a disordered and highly variable C-terminal tail (Figure 2) [29]. The SBDβ can accommodate exposed hydrophobic segments in nonnative polypeptides with its central hydrophobic cleft [29]. Mutations in the central hydrophobic cleft are often fatal for chaperone activity of Hsp70 [30]. Some mutations in the β6/β7 region of the Hsp70 substrate-binding domain are defective in prion propagation and heat-shock phenotypes in *Saccharomyces cerevisiae* [31,32]. In eukaryotic cytosolic and nuclear Hsp70 members, the C-terminal tail frequently ends with a conserved tetratricopeptide-repeat-domain-interaction EEVD motif, facilitating interactions with cochaperones [33,34]. In the *E. coli* Hsp70 homologue, DnaK, a ~15-residue motif in the C-terminal tail, can directly bind to misfolded protein substrates and enhance the protein refolding efficiency of DnaK [35]. In the *Saccharomyces cerevisiae* Hsp70 homolog, Ssa1, the 20-residue GGAP motif in the C-terminal tail, contributes to cell tolerance to temperature and cell-wall damage stress [36].

The conformation and function of Hsp70 are tightly related to allosteric mechanisms and binding of nucleotide and substrate [37]. In ADP-bound or nucleotide-free states, the two domains of Hsp70 remain separate as reflected by the NMR structure of DnaK [26]. ATP binding induces docking of the NBD and SBD with the association of the NBD and SBDꞵ, together with separation of the SBDα and SBDꞵ [38,39,40], so that the detached SBDα leans on the NBD [39,40]. The ATP-bound state also contains different functional states in equilibrium, such as a restrained state, which restricts ATP hydrolysis and blocks the client, and a stimulated state, which hydrolyzes ATP rapidly and binds the client with rapid on-and-off kinetics for DnaK [41]. The conformations between NBD and SBD show more obvious heterogeneity in human cytosolic stress-inducible Hsp70 (HspA1A); in the ADP-bound or nucleotide-free states, the NBD-SBD docked fraction is also observed in single-molecule fluorescence resonance energy transfer (smFRET) and NMR studies [38,42]. Generally, ATP binding and docking of the two domains tends to result in low substrate affinity and low ATPase activity [39]. Substrate binding to ATP-bound Hsp70 can change the equilibrium between the restrained state and the stimulated state and disrupt the interaction between the NBD and SBDꞵ, leading to undocking of the NBD and SBD and facilitating ATP hydrolysis [43]. ADP-bound Hsp70 has high affinity for substrates [43]. ATP-ADP exchange in the NBD restores Hsp70 to the ATP-bound state and facilitates the release of bound substrate [38]. ATP hydrolysis and ATP-ADP exchange in the NBD, and binding and release of substrates in the SBD, are coupled to form the functional cycle of Hsp70, which promotes protein folding and/or provides protection for clients by repeated temporary interactions. Hsp40 proteins trigger undocking of the NBD and SBDꞵ to stimulate ATP hydrolysis by Hsp70 and transfer substrates to Hsp70, and NEFs accelerate ATP-ADP exchange in the NBD by prompting ADP release [37,44,45,46]. Different Hsp40s and NEFs specifically tune this cycle, and the number of cochaperones has been observed to increase with evolution to guide Hsp70 to wider and more complex functions and regulatory mechanisms [20,21,47]. The functions of Hsp70 members are further extended by linking with other components of the proteostasis network such as other chaperones, including small heat shock proteins (sHSPs), Hsp60, Hsp90 and Hsp100, and protein degradation systems, including the ubiquitin–proteasome system (UPS) and chaperone-mediated autophagy (CMA) [21,48,49,50,51].

## 3. Post-Translational Modifications of Hsp70 under Oxidative Stress

ROS not only directly oxidize cysteine thiols in Hsp70 proteins but also cause covalent modification by production of lipoperoxides, altering the structure and chaperone activity of Hsp70 and affecting related cellular processes. Oxidation of extracellular Hsp70 alters its structure and signaling effects on macrophage function and viability, leading to lower proliferation, lower phagocytic activity and reduced TNF-α release [53]. These PTMs often inactivate the foldase activity of Hsp70 and change interaction with Hsp70 to modulate the activity of its clients and related signaling pathways. Thus, Hsp70 members can also act as redox sensors to transfer redox signals. For example, yeast Hsp70 Ssa1 can act as a sensor of thiol-reactive compounds, which probably involves modification of Cys264 and Cys303, activating Hsf1-mediated cytoprotection [54].

### 3.1. Cysteine Oxidation of Hsp70 and Redox Homeostasis

Most Hsp70 members contain at least one Cys residue, but different Hsp70 members vary in their distributions of Cys residues (Table 2). The number of Cys residues in Hsp70 increases with evolution, indicating the importance of cysteines for the function of Hsp70 to fit increasingly complex cellular environments (Table 2). These Cys residues often undergo different modifications under oxidative stress conditions to link Hsp70 with redox homeostasis (Table S1). Cys residues in Hsp70 generally show modest reactivity in global profiling of the cysteinome [55]. Over the past twenty years development of methods for enrichment of Cys-modified proteins and mass spectrometry (MS) detection has allowed extensive identification of cysteine modifications in Hsp70 members (Table S1), which have been summarized in databases for protein cysteine modifications [56,57,58,59,60]. Once cysteine modifications have been identified, the next goal is to identify the effects of cysteine modifications on the structure and function of Hsp70 and the physiological significance of these changes.

**Figure 3:**
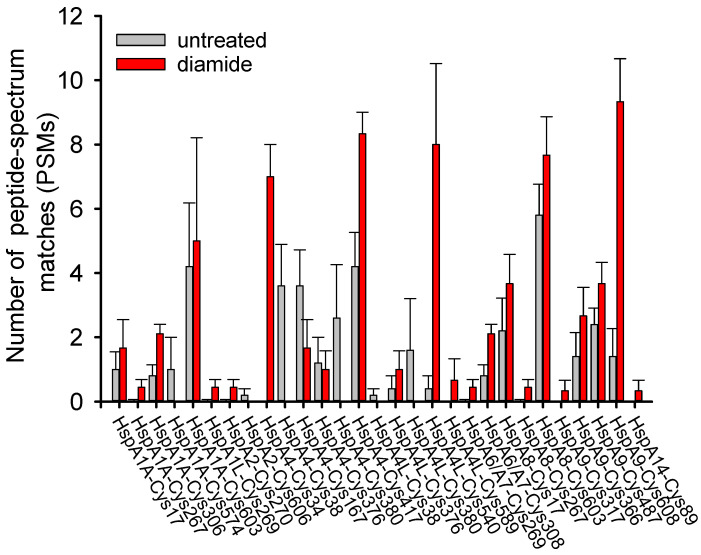
Glutathionylation of the cysteine residues of Hsp70 family members in HeLa cells with or without diamide treatment detected by mass spectrometry. Figure adapted from ref. [52].

Cysteine modifications can interconvert, and different cysteine modifications are often detected at the same cysteine residue [61]. Glutathionylation, sulfenic modification, S-nitrosylation and S-sulfhydration of multiple Hsp70 members are frequently identified by MS in a variety of organisms (Table S1). Most human Hsp70 members containing reactive Cys residues have been detected as undergoing cysteine modifications, suggesting that they can sense redox change and participate in regulation of redox homeostasis (Table S1, Figure 3). Among these members, HspA1A/B, HspA4, HspA4L, HspA8 and HspH1 are the most frequently detected human Hsp70 homologues when screening for cysteine modifications (Table S1), possibly due to their higher cysteine reactivity or higher protein levels in cells.

**Figure 4:**
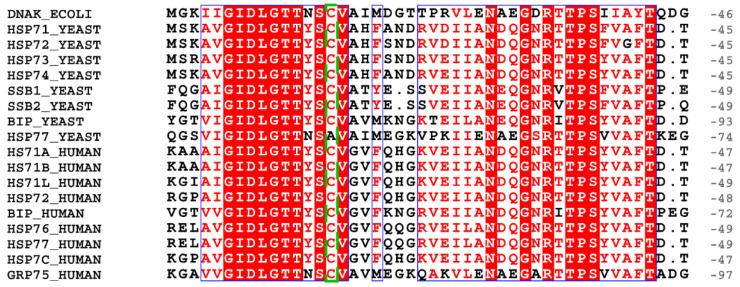
The most conserved Cys residue in Hsp70 homologues. The conserved Cys is indicated by the green dashed box. In each line the last number indicates the position of the last residue in the corresponding Hsp70 homologue.

*E. coli* DnaK only has one Cys residue, Cys15, which lies on the β-sheet surface of the IA subdomain of the NBD. The cysteine at this position is highly conserved among Hsp70 family members, suggesting its functional importance (Figure 4, corresponding to Cys17 in Figure 2). Thiol reactivity of this cysteine is affected by nucleotides and substrate binding and thus is closely related with allostery of DnaK, and only the apo state of DnaK favors glutathionylation [62]. Glutathionylation has a dramatic effect on the structure and function of DnaK, resulting in altered allostery of DnaK including decreased ATPase activity and substrate-binding ability [62]. Glutathionylated DnaK also loses the ability to cooperate with its cochaperones DnaJ and GrpE to promote refolding of luciferase [62]. However, deglutathionylation can fully restore the structure and function of DnaK, and so the alteration caused by glutathionylation is fully reversible [62]. Thus, glutathionylation can turn off chaperone activity of DnaK under oxidative stress conditions, and this activity is recovered under reducing conditions. Chaperone activity of Hsp70 is related to suppression of activation of some proteins, such as heat shock response (HSR) transcription factors. In *E. coli*, binding of sigma32 to DnaK facilitates its degradation to repress the HSR under normal conditions and disabling of DnaK caused by glutathionylation can contribute to prolonging the lifetime of sigma32 and its activation of the HSR [63,64]. Thus, glutathionylation of DnaK not only protects DnaK from irreversible oxidative damage but also provides a link between oxidative stress and the HSR. Under oxidative heat treatment, H_2_O_2_ causes S-sulfenation, S-sulfination and S-sulfonation of a proportion of the cellular DnaK, resulting in its inactivation [65].

Human HspA1A contains five Cys residues: Cys17, Cys267 and Cys306, located in the NBD, and Cys574 and Cys603, located in the SBDα (Figure 2). All five Cys residues have the possibility to undergo glutathionylation in untreated or diamide-treated HeLa cells (Figure 3) [52]. Nucleotides inhibit modification of Cys residues in the NBD but not in the SBDα, and binding of peptide substrate has no effect on the activity of these cysteine residues of HspA1A [52]. Glutathionylation of Cys574 and Cys603, located in the SBDα, causes unfolding of the α helix bundle of the SBDα, and the unfolded region mimics the substrate to bind to and block the substrate-binding site in the SBDꞵ, resulting in promotion of intrinsic ATPase activity and decreased binding of external substrates, including heat shock transcription factor Hsf1 [52]. Similar to glutathionylation of DnaK, glutathionylation of HspA1A is fully reversible, allowing its structure and function to be fully restored [52]. Thus, glutathionylation and deglutathionylation of HspA1A caused by ROS followed by restoration of reducing conditions can act as a switch to turn the chaperone activity of HspA1A off and on and to transfer redox signals to its clients. The cysteine activities of the five Cys residues are different, for example Cys603 is more active than Cys574, and so can act as a redox sensor in HspA1A [52]. S-nitrosylation of Cys17, Cys306, Cys574 and Cys603 in HspA1A upon GSNO treatment or NO signaling and S-sulfhydration of Cys306 and Cys574 in HspA1A upon hydrogen sulfide (H_2_S) signaling have also been identified [66,67,68,69,70,71].

Human HspA8, which undertakes more housekeeping chaperone functions, including assisting de novo folding of newly synthesized peptides and CMA, shares 86% identity with stress-inducible HspA1A and has four Cys residues at the same positions as HspA1A but lacks Cys306. HspA1A and HspA8 are mainly located in the cytosol, nucleus, cell membrane and extracellular exosomes [25]. Expression of *HSPA8* is constitutive and only slightly induced upon stress, while expression of *HSPA1A* varies in different tissues and is dramatically induced upon stress [25]. All four Cys residues in HspA8 have been detected as undergoing glutathionylation (Figure 3) [52,58]. However, HspA8 has much lower cysteine reactivity than HspA1A, indicating their different redox sensitivity and functional regulation [52]. The redox-active compound methylene blue (MB) cannot cause modification of Cys residues of HspA8 but can cause S-sulfenation of Cys306 in HspA1A, leading to exposure and oxidation of Cys267, resulting in decreased ATPase activity of HspA1A [72]. Thus, Cys306 distinguishes the different redox sensing of HspA1A and HspA8. The different sensitivity to redox and cysteine modifications of HspA1A and HspA8 may be related to their different functions under stress. S-sulfenation of Cys17, Cys574 and Cys603 in HspA8 and S-sulfinylation of Cys603 in HspA8 upon H_2_O_2_ treatment have also been detected by MS [73,74,75]. Expression of neuronal nitric oxide synthase (nNOS/NOS1) in SH-SY5Y cells releases nitric oxide (NO) and leads to S-nitrosylation of Cys17, Cys574 and Cys603 in HspA8 [76]. However, the regulation effect and physiological significance of S-nitrosylation of HspA8 is still unknown. Upon hydrogen sulfide (H_2_S) signaling, Cys574 and Cys603 in HspA8 also undergo S-sulfhydration [70,71].

In the ER, oxidative folding of proteins often generates ROS as a by-product. Yeast ER Hsp70 BiP (Kar2), like *E. coli* DnaK, has only one Cys residue, Cys63, which corresponds to the highly conserved Cys15 in DnaK (BiP has an extra N-terminal signal sequence) (Figure 4). Glutathionylation of yeast BiP is detected during ER oxidative stress and mediated by the cellular GSSG/GSH ratio, which is often measured to reflect the cellular redox status [77,78]. Glutathionylation decouples ATPase and peptide-binding activities of BiP, turning BiP from an ATP-dependent foldase into an ATP-independent holdase, which helps prevent protein aggregation [77]. The effect of glutathionylation on chaperone activity of BiP is also fully reversible upon removal of glutathionylation [77]. Glutathionylation promotes cell proliferation during oxidative stress, possibly due to the enhanced holdase activity of glutathionylated BiP. During oxidative stress, H_2_O_2_ also causes S-sulfenation of BiP, which leads to enhanced holdase activity of BiP to prevent protein aggregation, similar to the effects of glutathionylation. S-sulfenation of BiP can be converted into glutathionylation by reacting with GSH or can be converted into irreversible S-sulfination and/or S-sulfonation by further oxidation with H_2_O_2_ [77,79]. Thus, glutathionylation of BiP may also serve to prevent irreversible oxidation of BiP and preserve the capacity of BiP to undergo Cys reduction. Substituting the Cys residue in BiP with other amino acids to mimic unmodified or oxidized BiP results in decreased or increased cell viability in the presence of oxidants, respectively [80]. This indicates that levels of oxidized BiP can be tuned to maintain ER folding homeostasis under a range of redox conditions.

### 3.2. Covalent Modifications of Hsp70 and Redox Homeostasis

Oxidative stress often causes oxidation of lipids and overproduction of 4-hydroxynonenal (4-HNE), which is derived from peroxidation of polyunsaturated fatty acids [81]. 4-HNE is a highly reactive electrophile and can react with proteins, phospholipids and nucleic acids [81]. It can covalently modify His, Cys and Lys residues, causing functional impairment of proteins, leading to inhibition of enzyme activity and induction of apoptosis [82,83,84]. Thus, 4-HNE can act as a second messenger of free radicals and a growth regulatory factor [82,83,84]. The antibiotic plakortin can generate ROS and 4-HNE as products of lipoperoxides [85]. In plakortin-treated *Plasmodium falciparum*, 4-HNE-modified PfHsp70-1 and PfHsp70-2 (the BiP analogue) have been identified [85]. 4-HNE modification of Cys267 in rat Hsp72 was identified and found to decrease affinity of Hsp72 for ATP and reduce its luciferase refolding activity [86]. In a mouse model of alcoholic liver disease (ALD), Grp78 (a BiP analogue) undergoes modification by 4-HNE at some Lys and His residues within the NBD [87]. The 4-HNE modification decreases ATP binding and ATPase activity of Grp78 [87]. In human retinal pigment epithelial cells (ARPE-19), 4-HNE-modified Hsp70 cannot protect its client XIAP, resulting in promotion of XIAP degradation and apoptosis [88].

## 4. Protection Effect and Upregulated Expression of Hsp70 under Oxidative Stress

Hsp70 can counteract oxidative stress damage to proteins by inhibiting aggregation and/or facilitating degradation of oxidized proteins, as well as inducing the expression of key antioxidant enzymes such as SOD1 and CAT and adjusting the activity of antioxidant enzymes [15,89,90,91,92]. Overexpression of *HSP70* in the muscle of mice antagonizes the aging-related increase in protein cysteine modifications including carbonylation, oxidation and formation of disulfide bonds [93]. Overexpression of mitochondrial *HSP70/Hsp75* in rat brain protects mitochondria and results in the marked reduction in free radical generation [94]. Under oxidative stress, HspA1A and HspA8 can mediate the C-terminus of Hsp70-interacting protein (CHIP, also known as STUB1) to recognize and ubiquitinate ROS-stressed peroxisomes, resulting in their turnover by autophagy [95]. Overexpression of *HSP70* suppresses ROS production from mitochondria in human lung microvascular endothelial cells (HLMVEC) exposed to bacterial toxins [96]. The *HSPA1B* rs1061581 polymorphism (1267 A/G), which is linked to the risk of developing multiple sclerosis, is related to ROS levels and has a role in the variation in *HSPA1B* expression levels under oxidative stimulus [97].

Oxidative stress can activate the HSR and Kelch-like ECH-associated protein 1 (Keap1)/Nrf2 pathway to upregulate expression of *HSP70*, and the increased protein level of Hsp70 protects cells against oxidative-stress-related damage [15,98,99]. However, different types of oxidative stress differ in their capacity to induce the HSR and elevate expression of *HSP70*, and the same oxidative stimulus may lead to distinct stress responses in different cells [100,101,102]. Oxidative-stress induced by 15-deoxy-Δ12,14-prostaglandin J2 (15d-PGJ2) enhances *HSP70* expression to exert anti-inflammatory effects [103]. Under mild oxidative stress conditions, expression of *HSPA1A/B* is upregulated in the mammalian neuronal cell line HT22 [90,104]. In the early phase after oxidative stress, HspA1A/B binds to partially unfolded oxidized proteins to prevent their aggregation [104]. Then, HspA1A/B facilitates the degradation of bound oxidized proteins by 20S proteasomes [104]. Expression of HspA8 is not affected by oxidative stress, and HspA8 has no effect on removal of proteins damaged by oxidation by the 20S proteasome [104]. Thus, HspA1A/B but not HspA8 mediates degradation of damaged proteins by the UPS under oxidative stress conditions [104], while HspA8-facilitated CMA is also activated by oxidative stress to facilitate degradation of oxidized proteins [105,106].

## 5. Hsp70 Participates in Multiple Redox-Related Signaling Pathways

Hsp70 and redox homeostasis are linked by multiple signaling pathways including inositol-4,5-bisphosphate 3-kinase (PI3K)/protein kinase B (PKB, Akt)/Hsp70, Janus kinase 2 (JAK2)/signal transducer and activator of transcription 3 (STAT3)/Hsp70, and Keap1/Nrf2/Hsf1/Hsp70. Expression or activity of Hsp70 is often modulated by the related signaling pathways [107,108,109].

SOD2 protects cells against mitochondrial oxidative damage [110]. HspA1A can bind to SOD2 to prevent its aggregation and control its CHIP-mediated degradation or import it into mitochondria [107,111]. CHIP cooperates with HspA1A by binding to the C-terminus EEVD motif and the SBDα of HspA1A [112]. PI3K/Akt signaling is crucial for receiving input from the cell membrane and mitochondria [113]. Akt1 is the downstream kinase of PI3K and a client of HspA1A. Akt1 binds to the SBDα of HspA1A and phosphorylates the C-terminus Ser631 of HspA1A, resulting in decoupling of HspA1A and CHIP and inhibition of the degradation of SOD2 [107]. Then, the import of SOD2 into mitochondria is promoted. As a negative feedback loop, the resulting decrease in oxidative stress activation permits phosphatase 2C (PP2C) to dephosphorylate HspA1A to promote degradation of SOD2 [107]. Thus, Hsp70 phosphorylation involved in the PI3K/Akt/Hsp70 signaling pathway contributes to redox homeostasis.

The oncogenic signaling pathway JAK2/STAT3 plays crucial roles in regulating apoptosis, proliferation, differentiation, and the inflammatory response and participates in the occurrence and development of various tumors [114]. AG490 is an inhibitor of the JAK2/STAT3 signaling pathway [115]. It was found that H_2_O_2_-induced time-dependent increase in Hsp70 protein expression in vascular smooth muscle cells is inhibited by pretreatment with AG-490, suggesting that ROS can regulate *HSP70* expression via the JAK2/STAT3 signaling pathway [116]. It was also found that both *HSP70* RNAi and AG490 can increase ROS levels in Burkitt’s lymphoma Raji cells and reduce the activity of SOD and GPX, suggesting that inhibiting Hsp70 or the JAK2/STAT3 pathway may induce oxidative stress of Raji cells [108]. Therefore, the JAK2/STAT3 signaling pathway may contribute to redox homeostasis by modulating the expression of *HSP70*.

The Keap1/Nrf2 pathway is a thiol-based sensor–effector for maintaining redox homeostasis [16]. Keap1 acts as a cysteine-thiol-rich sensor of redox status, and Nrf2 is a transcription factor for some cytoprotective genes including some enzymatic antioxidants and Hsp70 [16]. The Keap1/Nrf2 complex facilitates degradation of Nrf2 and represses the activity of Nrf2 [16]. ROS or electrophiles can cause cysteine modification of Keap1, which contains multiple active thiols, to weaken the interaction between Keap1 and Nrf2 and promote the entrance of Nrf2 into the cell nucleus and activation of Nrf2 [16,117]. Hsf1 is an important transcription factor for genes related to HSR. Hsp90, Hsp70 and Hsp40 cooperate to inactivate Hsf1 by binding to it and preventing it from entering the cell nucleus, and they are also transcription targets of Hsf1, forming a negative feedback regulatory loop [118,119]. Although the mechanism by which Hsf1 is activated has not been fully elucidated, activation of Hsf1 is thought to be related to stress damage of proteins and modification of HSPs or Hsf1 [117,120]. Both Nrf2 and Hsf1 are critical for adaptation and survival, and there is crosstalk between the two pathways through overlapping transcriptional targets including *HSP70* [99]. Some reports indicate that the Keap1/Nrf2 pathway is activated before the heat shock response upon oxidative or electrophilic stress [99,121,122]. When Hsf1 is mutated and therefore unable to activate the HSR, Nrf2 can be activated by heat shock to cause delayed upregulation of *HSP70*, suggesting the two pathways may compensate for each other [123]. Oxidative stress resulting from a lack of methionine induces *HSPA1A* expression through Nrf2 activation but not Hsf1 activation in HEK293 cells [109]. Both Hsf1 and Nrf2 pathways contribute to redox homeostasis and cooperate to promote a more reducing cellular environment.

## 6. Conclusions and Perspectives

Redox homeostasis relies on the balance between ROS generation and the antioxidation system. Hsp70 can buffer different kinds of stress including oxidative stress. Hsp70 members are important antioxidative components in eliminating damaged oxidized proteins and support antioxidative enzymes. Under oxidative stress, Hsp70 members will inevitably undergo oxidization, resulting in changes in their structure and chaperone activity. However, cysteine modifications often downregulate the chaperone activity of Hsp70, which is disadvantageous for dealing with protein damage due to oxidation. From this point of view, cysteine residues in Hsp70 are not conducive towards Hsp70 exerting its antioxidative functions. At the same time, only very few Hsp70 members do not contain any cysteine residues, and the number of cysteine residues in Hsp70 increases with evolution, suggesting important roles of cysteine residues in Hsp70. Hsp70 members can sense redox by cysteine modifications, then transfer redox signals by modulating the interaction between Hsp70 members and their clients. Hsp70 members are involved in cellular signaling due to the fact that their clients include key molecules in signaling pathways, including Hsf1 and Akt1. Cochaperones also guide Hsp70 members within different cellular processes, such as CHIP, acting as a link between Hsp70 and the UPS for degradation of Hsp70 clients. Thus, cysteine modifications of Hsp70 also change the fate of the clients by modifying the cooperation between Hsp70 and cochaperones. Therefore, cysteine modifications of Hsp70 may act to amplify the transfer of redox signaling to a broader range of proteins, achieving greater antioxidative potential in a shorter time and restoring the cellular environment by multiple pathways. As a consequence, Hsp70 proteins are expressed much more rapidly through different signaling pathways, and the newly synthesized Hsp70 proteins are not oxidized and have normal chaperone activity to deal with the damaged proteins that have accumulated during oxidative stress. Thus, combining an in-depth understanding of the basic principles of Hsp70 systems (including the relationship between the structure and function of Hsp70, the interaction between Hsp70 and its cochaperones, and the interaction between Hsp70 and its clients) and the extensive exploration of redox-related signaling pathways involving Hsp70 will lead to a fuller understanding of Hsp70 function in redox homeostasis.

## Figures and Tables

**Table 1:** Redox-regulated cellular signaling [5].

Cellular Processes	Critical Signaling Molecules Modified by ROS
proliferation and survival	mitogen-activated protein kinases (MAPKs), phosphatidylinositol-4,5-bisphosphate 3-kinase (PI3K), phosphatase and tensin homolog deleted on chromosome ten (PTEN) and protein tyrosine phosphatases
redox homeostasis	thioredoxin, peroxiredoxin, redox factor-1 (Ref-1) and Kelch-like ECH-associated protein 1 (Keap1)/nuclear factor erythroid 2-related factor 2 (Nrf2)
mitochondrial oxidative stress and aging	p66Shc
iron homeostasis	iron response element–iron regulatory protein (IRE-IRP) containing iron–sulfur cluster
DNA damage response	ataxia telangiectasia mutated (ATM)

**Table 2:** Cysteine residues in bacteria, yeast and human Hsp70 homologs.

Hsp70 Homolog	Cysteine Residues (Entry in UniProtKB)
*Escherichia coli* DnaK	Cys15 (P0A6Y8)
*Escherichia coli* HscA (Hsc66)	Cys315, Cys448 (P0A6Z1)
*Escherichia coli* HscC (Hsc62)	Cys236, Cys242, Cys261, Cys344, Cys360 (P77319)
*Saccharomyces cerevisiae* Ssa1 (cytosol)	Cys15, Cys264, Cys303 (P10591)
*Saccharomyces cerevisiae* Ssa2 (cytosol)	Cys15, Cys264, Cys303 (P10592)
*Saccharomyces cerevisiae* Ssa3 (cytosol)	Cys15, Cys304 (P09435)
*Saccharomyces cerevisiae* Ssa4 (cytosol)	Cys15, Cys304 (P22202)
*Saccharomyces cerevisiae* Ssb1 (cytosol)	Cys20, Cys435, Cys454 (P11484)
*Saccharomyces cerevisiae* Ssb2 (cytosol)	Cys20, Cys454 (P40150)
*Saccharomyces cerevisiae* Sse1 (cytosol Hsp110)	Cys142, Cys211, Cys228, Cys380, Cys484 (P32589)
*Saccharomyces cerevisiae* Sse2 (cytosol Hsp110)	Cys142, Cys211, Cys380, Cys484 (P32590)
*Saccharomyces cerevisiae* Ssz1 (cytosol)	Cys81, Cys86 (P38788)
*Saccharomyces cerevisiae* Ssc1 (mitochondria)	None (P0CS90)
*Saccharomyces cerevisiae* Ssc2 (Ssq1) (mitochondria)	Cys134 (Q05931)
*Saccharomyces cerevisiae* Ssc3 (Ecm10) (mitochondria)	None (P39987)
*Saccharomyces cerevisiae* Ssd1 (Kar2, BiP, Grp78) (ER)	Cys63 (P16474)
*Saccharomyces cerevisiae* Lhs1 (Grp170) (ER)	Cys520, Cys545, Cys547 (P36016)
Human HspA1A (Hsp72) (cytosol, nucleus, cell membrane, extracellular exosomes)	Cys17, Cys267, Cys306, Cys574, Cys603 (P0DMV8)
Human HspA1B (Hsp72) (cytosol, nucleus, extracellular exosomes)	Cys17, Cys267, Cys306, Cys574, Cys603 (P0DMV9)
Human HspA1L (cytosol, nucleus)	Cys19, Cys269, Cys308, Cys576, Cys605, Cys617, Cys622 (P34931)
Human HspA2 (cytosol, nucleus, cell membrane, extracellular exosomes)	Cys18, Cys191, Cys270, Cys577, Cys606 (P54652)
Human HspA4 (Apg2) (cytosol, extracellular exosome, mitochondrion, nucleus)	Cys13, Cys34, Cys38, Cys140, Cys146, Cys167, Cys213, Cys245, Cys270, Cys290, Cys310, Cys376, Cys380, Cys417, Cys779 (P34932)
Human HspA4L (Apg1, Osp94) (cytosol, nucleus)	Cys13, Cys34, Cys38, Cys140, Cys167, Cys213, Cys245, Cys270, Cys290, Cys310, Cys376, Cys380, Cys417, Cys421, Cys540, Cys589, Cys740, Cys782 (O95757)
Human HspA5 (BiP, Grp78) (ER, extracellular exosomes)	Cys41, Cys420 (P11021)
Human HspA6 (Hsp70B’) (cytosol, extracellular exosomes)	Cys19, Cys108, Cys269, Cys308, Cys387, Cys576, Cys605, Cys624 (P17066)
Human HspA7 (Hsp70B) (blood microparticles, extracellular exosomes)	Cys19, Cys108, Cys269, Cys308 (P48741)
human HspA8 (Hsc70, Hsc73) (cytosol, nucleus, cell membrane, extracellular exosomes)	Cys17, Cys267, Cys574, Cys603 (P11142)
Human HspA9 (Grp75, mt-Hsp70) (mitochondria, nucleus)	Cys66, Cys317, Cys366, Cys487, Cys608 (P38646)
Human HspA12A (extracellular exosomes, nucleus)	Cys80, Cys246, Cys502, Cys564, Cys621 (O43301)
Human HspA12B (endothelial cells, intracellular, blood plasma)	Cys36, Cys106, Cys250, Cys321, Cys365, Cys450, Cys570, Cys595, Cys610, Cys611, Cys626, Cys639 (Q96MM6)
Human HspA13 (Stch) (ER, extracellular exosomes, microsomes)	Cys43 (P48723)
Human HspA14 (Hsp60, Hsp70L1) (cytosol, membrane)	Cys10, Cys14, Cys89, Cys280, Cys293, Cys304, Cys311, Cys335, Cys394, Cys440, Cys492, Cys500 (Q0VDF9)
Human HspH1 (Hsp105, Hsp110) (microtubule, cytosol, extracellular region or secreted, nucleus)	Cys13, Cys34, Cys48, Cys140, Cys167, Cys213, Cys245, Cys270, Cys290, Cys310, Cys376, Cys380, Cys516, Cys650, Cys658, Cys796, Cys845 (Q92598)
Human Hyou1 (Grp170, Orp150) (ER, extracellular region or secreted)	Cys15, Cys240, Cys352, Cys805 (Q9Y4L1)

## Supplementary Materials

The following supporting information can be downloaded at: https://www.mdpi.com/article/10.3390/cells11050829/s1, Table S1: Cysteine modifications identified in Hsp70 members. References [124,125,126,127,128,129,130,131,132,133,134,135,136,137,138,139,140,141,142,143,144,145,146,147,148,149,150,151,152,153,154,155,156,157,158,159,160,161,162,163] were cited in the supplementary materials.
