# Supplementary material for: Hsp70 in Redox Homeostasis
Source: Cells. 2022 Feb 28;11(5):829. doi: 10.3390/cells11050829 (PMC8909019; doi:10.3390/cells11050829)
Supplement: Supplementary file 1 [file cells-11-00829-s001.zip › cells-1582682-supplementary.pdf]

Supporting Information for:

Review

Hsp70 in redox homeostasis

Hong Zhang [1,2*], Weibin Gong [1], Si Wu [1,2] and Sarah Perrett [1,2*]

[1] National Laboratory of Biomacromolecules, CAS Center for Excellence in Biomacromolecules, Institute of Biophysics, Chinese Academy of Sciences, 15 Datun Road, Chaoyang District, Beijing 100101, China. gongweibin@ibp.ac.cn (W.G.); wusi@ibp.ac.cn (S.W.)

[2] University of the Chinese Academy of Sciences, 19 Yuquan Road, Shijingshan District, Beijing 100049, China.

* Correspondence: zhangh@moon.ibp.ac.cn (H. Z.); sarah.perrett@cantab.net (S. P.)

Table S1. Cysteine modifications identified in Hsp70 members

	glutathionylation	sulfenic modification	S-nitrosylation	S-sulfhydration
Escherichia coli	DnaK [1]	DnaK [2]	DnaK [3]	
Salmonella typimurium	DnaK [4]			
Saccharomyces cerevisiae	BiP (Kar2) [5]	BiP (Kar2) [5,6]		
Chlamydomonas reinhardtii	Hsp70b [7]			
Arabidopsis thaliana		Hsp70-1 [8], Hsp70-2 [8], Hsp70-3 [8], Hsp70-4 [8], Hsp70-8 [8], Hsp70-9 [8], Hsp70-14 [8], Hsp70-15 [8], Hsp70-16 [8]	Hsp70-1 [9–11], Hsp70-2 [9–11], Hsp70-3 [9–11], Hsp70-4 [10,11], Hsp70-6 [9], Hsp70-7 [9], Hsp70-10 [10], Hsp70-14 [9], Hsp70-15 [9], Hsp70-16 [9], BiP1 [10], BiP2 [10]	Hsp70-1 [12], Hsp70-3 [12], Hsp70-6 [12]
Solanum lycopersicum			Hsc-1 [13,14], Hsc-2 [13,14], Hsc80 [14]	
Drosophila melanogaster	Hsc70-1 [15], Hsc70-3 [15], Hsc70-4 [15], Hsc70-5 [15], Hsp70Aa [15], Hsp70Ab [15], Hsp70Ba [15],			

	Hsp70Bb [15], Hsp70Bbb [15], Hsp70Bc [15]			
Mus musculus	Hspa4 [16–20], Hspa8 [4,16–18,20,21], Hsph1[16–20]		Hspa1a/b [22], Hspa4 [19,22–27], Hspa8 [19,21–24,26–33], Hsph1 [19,23,25,26,29,34]	
Rattus norvegicus			Hspa4 [24,35], Hspa8 [24,36], Hsph1[24,36]	
Bos taurus			HspA1A/B [37,38], HspA6 [37]	
Homo sapiens	HspA1A/B [16,39–41], HspA1L [39], HspA2 [39], HspA4 [16,39,40], HspA4L [16,39,40], HspA5 (the ER-resident Hsp70 BiP) [42], HspA6 [39,40], HspA7 [39,40], HspA8 [39,42,43], HspA9 (the mitochondrial Hsp70 Mortalin) [39], HspA14 [39], HspH1 [16,39,40]	HspA1A/B [44], HspA4 [45], HspA4L [45], HspA8 [45,46], HspH1 [45,46]	HspA1A/B [30,47–49], HspA1L [50], HspA4 [24,30,37,47,51,52], HspA4L [30,37,47,52], HspA6 [30,50], HspA7 [30], HspA8 [24,30,37,47,50–53], HspA9 [48,49], HspA12A [47], HspA14 [47], HspH1 [30,37,47,54–56]	HspA1A/B [57,58], HspA4 [57,58], HspA4L [57], HspA5 [57] HspA6 [57,58], HspA7 [57,58], HspA8 [57,58], HspH1 [57]

References

1. Zhang, H., Yang, J., Wu, S., Gong, W., Chen, C., and Perrett, S., Glutathionylation of the Bacterial Hsp70 Chaperone DnaK Provides a Link between Oxidative Stress and the Heat Shock Response. *J Biol Chem*, **2016**, *291(13)*, 6967-6981, DOI: 10.1074/jbc.M115.673608.
2. Winter, J., Linke, K., Jatzek, A., and Jakob, U., Severe oxidative stress causes inactivation of DnaK and activation of the redox-regulated chaperone Hsp33. *Mol Cell*, **2005**, *17(3)*, 381-92, DOI: 10.1016/j.molcel.2004.12.027.
3. Wiktorowicz, J.E., Stafford, S., Rea, H., Urvil, P., Soman, K., Kurosky, A., Perez-Polo, J.R., and Savidge, T.C., Quantification of cysteinyl S-nitrosylation by fluorescence in unbiased proteomic studies. *Biochemistry*, **2011**, *50(25)*, 5601-14, DOI: 10.1021/bi200008b.
4. Ansong, C., Wu, S., Meng, D., Liu, X., Brewer, H.M., Deatherage Kaiser, B.L., Nakayasu, E.S., Cort, J.R., Pevzner, P., Smith, R.D., Heffron, F., Adkins, J.N., and Pasa-Tolic, L.,

Top-down proteomics reveals a unique protein S-thiolation switch in Salmonella Typhimurium in response to infection-like conditions. *Proc Natl Acad Sci U S A*, **2013**, *110(25)*, 10153-8, DOI: 10.1073/pnas.1221210110.

5. Wang, J. and Sevier, C.S., Formation and Reversibility of BiP Protein Cysteine Oxidation Facilitate Cell Survival during and post Oxidative Stress. *J Biol Chem*, **2016**, *291(14)*, 7541-7757, DOI: 10.1074/jbc.M115.694810.
6. Wang, J., Pareja, K.A., Kaiser, C.A., and Sevier, C.S., Redox signaling via the molecular chaperone BiP protects cells against endoplasmic reticulum-derived oxidative stress. *Elife*, **2014**, *3*, e03496, DOI: 10.7554/eLife.03496.
7. Michelet, L., Zaffagnini, M., Vanacker, H., Le Marechal, P., Marchand, C., Schroda, M., Lemaire, S.D., and Decottignies, P., In vivo targets of S-thiolation in Chlamydomonas reinhardtii. *J Biol Chem*, **2008**, *283(31)*, 21571-8, DOI: 10.1074/jbc.M802331200.
8. Huang, J., Willems, P., Wei, B., Tian, C., Ferreira, R.B., Bodra, N., Martínez Gache, S.A., Wahni, K., Liu, K., Vertommen, D., Gevaert, K., Carroll, K.S., Van Montagu, M., Yang, J., Van Breusegem, F., and Messens, J., Mining for protein S-sulfenylation in Arabidopsis uncovers redox-sensitive sites. *Proc Natl Acad Sci U S A*, **2019**, *116(42)*, 21256-21261, DOI: 10.1073/pnas.1906768116.
9. Hu, J., Huang, X., Chen, L., Sun, X., Lu, C., Zhang, L., Wang, Y., and Zuo, J., Site-specific nitrosoproteomic identification of endogenously S-nitrosylated proteins in Arabidopsis. *Plant Physiol*, **2015**, *167(4)*, 1731-46, DOI: 10.1104/pp.15.00026.
10. Chaki, M., Shekariesfahlan, A., Ageeva, A., Mengel, A., von Toerne, C., Durner, J., and Lindermayr, C., Identification of nuclear target proteins for S-nitrosylation in pathogen-treated Arabidopsis thaliana cell cultures. *Plant Sci*, **2015**, *238*, 115-26, DOI: 10.1016/j.plantsci.2015.06.011.
11. Fares, A., Rossignol, M., and Peltier, J.B., Proteomics investigation of endogenous S-nitrosylation in Arabidopsis. *Biochem Biophys Res Commun*, **2011**, *416(3-4)*, 331-6, DOI: 10.1016/j.bbrc.2011.11.036.
12. Aroca, A., Benito, J.M., Gotor, C., and Romero, L.C., Persulfidation proteome reveals the regulation of protein function by hydrogen sulfide in diverse biological processes in Arabidopsis. *J Exp Bot*, **2017**, *68(17)*, 4915-4927, DOI: 10.1093/jxb/erx294.
13. Gong, B. and Shi, Q., Identifying S-nitrosylated proteins and unraveling S-nitrosoglutathione reductase-modulated sodic alkaline stress tolerance in Solanum lycopersicum L. *Plant Physiol Biochem*, **2019**, *142*, 84-93, DOI: 10.1016/j.plaphy.2019.06.020.
14. Gong, B., Yan, Y., Zhang, L., Cheng, F., Liu, Z., and Shi, Q., Unravelling GSNOR-Mediated S-Nitrosylation and Multiple Developmental Programs in Tomato Plants. *Plant Cell Physiol*, **2019**, *60(11)*, 2523-2537, DOI: 10.1093/pcp/pcz143.
15. Feng, S., Chen, Y., Yang, F., Zhang, L., Gong, Y., Adilijiang, G., Gao, Y., and Deng, H., Development of a Clickable Probe for Profiling of Protein Glutathionylation in the Central Cellular Metabolism of E. coli and Drosophila. *Chem Biol*, **2015**, *22(11)*, 1461-1469, DOI: 10.1016/j.chembiol.2015.09.012.
16. Chen, Y.J., Lu, C.T., Lee, T.Y., and Chen, Y.J., dbGSH: a database of S-glutathionylation. *Bioinformatics*, **2014**, *30(16)*, 2386-2388, DOI: 10.1093/bioinformatics/btu301.
17. Su, D., Gaffrey, M.J., Guo, J., Hatchell, K.E., Chu, R.K., Clauss, T.R., Aldrich, J.T., Wu,

S., Purvine, S., Camp, D.G., Smith, R.D., Thrall, B.D., and Qian, W.J., Proteomic identification and quantification of S-glutathionylation in mouse macrophages using resin-assisted enrichment and isobaric labeling. *Free Radic Biol Med*, **2014**, *67*, 460-70, DOI: 10.1016/j.freeradbiomed.2013.12.004.
18. Duan, J., Kodali, V.K., Gaffrey, M.J., Guo, J., Chu, R.K., Camp, D.G., Smith, R.D., Thrall, B.D., and Qian, W.J., Quantitative Profiling of Protein S-Glutathionylation Reveals Redox-Dependent Regulation of Macrophage Function during Nanoparticle-Induced Oxidative Stress. *ACS Nano*, **2016**, *10(1)*, 524-38, DOI: 10.1021/acsnano.5b05524.
19. Fowler, N.J., Blanford, C.F., de Visser, S.P., and Warwicker, J., Features of reactive cysteines discovered through computation: from kinase inhibition to enrichment around protein degrons. *Sci Rep*, **2017**, *7(1)*, 16338, DOI: 10.1038/s41598-017-15997-z.
20. VanHecke, G.C., Yapa Abeywardana, M., Huang, B., and Ahn, Y.H., Isotopically Labeled Clickable Glutathione to Quantify Protein S-Glutathionylation. *Chembiochem*, **2020**, *21(6)*, 853-859, DOI: 10.1002/cbic.201900528.
21. Gould, N.S., Evans, P., Martínez-Acedo, P., Marino, S.M., Gladyshev, V.N., Carroll, K.S., and Ischiropoulos, H., Site-Specific Proteomic Mapping Identifies Selectively Modified Regulatory Cysteine Residues in Functionally Distinct Protein Networks. *Chem Biol*, **2015**, *22(7)*, 965-75, DOI: 10.1016/j.chembiol.2015.06.010.
22. Su, D., Shukla, A.K., Chen, B., Kim, J.S., Nakayasu, E., Qu, Y., Aryal, U., Weitz, K., Clauss, T.R., Monroe, M.E., Camp, D.G., 2nd, Bigelow, D.J., Smith, R.D., Kulkarni, R.N., and Qian, W.J., Quantitative site-specific reactivity profiling of S-nitrosylation in mouse skeletal muscle using cysteinyl peptide enrichment coupled with mass spectrometry. *Free Radic Biol Med*, **2013**, *57*, 68-78, DOI: 10.1016/j.freeradbiomed.2012.12.010.
23. Chen, Y.J., Ku, W.C., Lin, P.Y., Chou, H.C., Khoo, K.H., and Chen, Y.J., S-alkylating labeling strategy for site-specific identification of the s-nitrosoproteome. *J Proteome Res*, **2010**, *9(12)*, 6417-39, DOI: 10.1021/pr100680a.
24. Lee, T.Y., Chen, Y.J., Lu, C.T., Ching, W.C., Teng, Y.C., Huang, H.D., and Chen, Y.J., dbSNO: a database of cysteine S-nitrosylation. *Bioinformatics*, **2012**, *28(17)*, 2293-5, DOI: 10.1093/bioinformatics/bts436.
25. Zaręba-Kozioł, M., Szwajda, A., Dadlez, M., Wysłouch-Cieszyńska, A., and Lalowski, M., Global analysis of S-nitrosylation sites in the wild type (APP) transgenic mouse brain-clues for synaptic pathology. *Mol Cell Proteomics*, **2014**, *13(9)*, 2288-305, DOI: 10.1074/mcp.M113.036079.
26. Ibáñez-Vea, M., Huang, H., Martínez de Morentin, X., Pérez, E., Gato, M., Zuazo, M., Arasanz, H., Fernández-Irigoyen, J., Santamaría, E., Fernandez-Hinojal, G., Larsen, M.R., Escors, D., and Kochan, G., Characterization of Macrophage Endogenous S-Nitrosoproteome Using a Cysteine-Specific Phosphonate Adaptable Tag in Combination with TiO(2) Chromatography. *J Proteome Res*, **2018**, *17(3)*, 1172-1182, DOI: 10.1021/acs.jproteome.7b00812.
27. Kohr, M.J., Aponte, A.M., Sun, J., Wang, G., Murphy, E., Gucek, M., and Steenbergen, C., Characterization of potential S-nitrosylation sites in the myocardium. *Am J Physiol Heart Circ Physiol*, **2011**, *300(4)*, H1327-35, DOI: 10.1152/ajpheart.00997.2010.
28. Sun, M.A., Wang, Y., Cheng, H., Zhang, Q., Ge, W., and Guo, D., RedoxDB--a curated

database for experimentally verified protein oxidative modification. *Bioinformatics*, **2012**, *28(19)*, 2551-2552, DOI: 10.1093/bioinformatics/bts468.

29. Doulias, P.T., Tenopoulou, M., Greene, J.L., Raju, K., and Ischiropoulos, H., Nitric oxide regulates mitochondrial fatty acid metabolism through reversible protein S-nitrosylation. *Sci Signal*, **2013**, *6(256)*, rs1, DOI: 10.1126/scisignal.2003252.
30. Chung, H.S., Murray, C.I., Venkatraman, V., Crowgey, E.L., Rainer, P.P., Cole, R.N., Bomgarden, R.D., Rogers, J.C., Balkan, W., Hare, J.M., Kass, D.A., and Van Eyk, J.E., Dual Labeling Biotin Switch Assay to Reduce Bias Derived From Different Cysteine Subpopulations: A Method to Maximize S-Nitrosylation Detection. *Circ Res*, **2015**, *117(10)*, 846-857, DOI: 10.1161/circresaha.115.307336.
31. Kohr, M.J., Aponte, A., Sun, J., Gucek, M., Steenbergen, C., and Murphy, E., Measurement of S-nitrosylation occupancy in the myocardium with cysteine-reactive tandem mass tags: short communication. *Circ Res*, **2012**, *111(10)*, 1308-12, DOI: 10.1161/circresaha.112.271320.
32. Qu, Z., Meng, F., Bomgarden, R.D., Viner, R.I., Li, J., Rogers, J.C., Cheng, J., Greenlief, C.M., Cui, J., Lubahn, D.B., Sun, G.Y., and Gu, Z., Proteomic quantification and site-mapping of S-nitrosylated proteins using isobaric iodoTMT reagents. *J Proteome Res*, **2014**, *13(7)*, 3200-11, DOI: 10.1021/pr401179v.
33. Gu, L. and Robinson, R.A., High-throughput endogenous measurement of S-nitrosylation in Alzheimer's disease using oxidized cysteine-selective cPILOT. *Analyst*, **2016**, *141(12)*, 3904-15, DOI: 10.1039/c6an00417b.
34. Raju, K., Doulias, P.T., Evans, P., Krizman, E.N., Jackson, J.G., Horyn, O., Daikhin, Y., Nissim, I., Yudkoff, M., Nissim, I., Sharp, K.A., Robinson, M.B., and Ischiropoulos, H., Regulation of brain glutamate metabolism by nitric oxide and S-nitrosylation. *Sci Signal*, **2015**, *8(384)*, ra68, DOI: 10.1126/scisignal.aaa4312.
35. Smith, J.G., Aldous, S.G., Andreassi, C., Cuda, G., Gaspari, M., and Riccio, A., Proteomic analysis of S-nitrosylated nuclear proteins in rat cortical neurons. *Sci Signal*, **2018**, *11(537)*, DOI: 10.1126/scisignal.aar3396.
36. Pan, K.T., Chen, Y.Y., Pu, T.H., Chao, Y.S., Yang, C.Y., Bomgarden, R.D., Rogers, J.C., Meng, T.C., and Khoo, K.H., Mass spectrometry-based quantitative proteomics for dissecting multiplexed redox cysteine modifications in nitric oxide-protected cardiomyocyte under hypoxia. *Antioxid Redox Signal*, **2014**, *20(9)*, 1365-81, DOI: 10.1089/ars.2013.5326.
37. Zhang, H.H., Lechuga, T.J., Chen, Y., Yang, Y., Huang, L., and Chen, D.B., Quantitative Proteomics Analysis of VEGF-Responsive Endothelial Protein S-Nitrosylation Using Stable Isotope Labeling by Amino Acids in Cell Culture (SILAC) and LC-MS/MS. *Biol Reprod*, **2016**, *94(5)*, 114, DOI: 10.1095/biolreprod.116.139337.
38. Xie, Y., Luo, X., Li, Y., Chen, L., Ma, W., Huang, J., Cui, J., Zhao, Y., Xue, Y., Zuo, Z., and Ren, J., DeepNitro: Prediction of Protein Nitration and Nitrosylation Sites by Deep Learning. *Genomics Proteomics Bioinformatics*, **2018**, *16(4)*, 294-306, DOI: 10.1016/j.gpb.2018.04.007.
39. Yang, J., Zhang, H., Gong, W., Liu, Z., Wu, H., Hu, W., Chen, X., Wang, L., Wu, S., Chen, C., and Perrett, S., S-Glutathionylation of human inducible Hsp70 reveals a regulatory mechanism involving the C-terminal α-helical lid. *J Biol Chem*, **2020**, *295(24)*, 8302-8324,

DOI: 10.1074/jbc.RA119.012372.
40. Chou, C.C., Chiang, B.Y., Lin, J.C., Pan, K.T., Lin, C.H., and Khoo, K.H., Characteristic tandem mass spectral features under various collision chemistries for site-specific identification of protein S-glutathionylation. *J Am Soc Mass Spectrom*, **2015**, *26(1)*, 120-32, DOI: 10.1007/s13361-014-1014-9.
41. Konstantinidis, D., Paletas, K., Koliakos, G., and Kaloyianni, M., The ambiguous role of the Na+-H+ exchanger isoform 1 (NHE1) in leptin-induced oxidative stress in human monocytes. *Cell Stress Chaperones*, **2009**, *14(6)*, 591-601, DOI: 10.1007/s12192-009-0110-4.
42. Fratelli, M., Gianazza, E., and Ghezzi, P., Redox proteomics: identification and functional role of glutathionylated proteins. *Expert Rev Proteomics*, **2004**, *1(3)*, 365-376, DOI: 10.1586/14789450.1.3.365.
43. Hoppe, G., Chai, Y.C., Crabb, J.W., and Sears, J., Protein s-glutathionylation in retinal pigment epithelium converts heat shock protein 70 to an active chaperone. *Exp Eye Res*, **2004**, *78(6)*, 1085-92, DOI: 10.1016/j.exer.2004.02.001.
44. Miyata, Y., Rauch, J.N., Jinwal, U.K., Thompson, A.D., Srinivasan, S., Dickey, C.A., and Gestwicki, J.E., Cysteine reactivity distinguishes redox sensing by the heat-inducible and constitutive forms of heat shock protein 70. *Chem Biol*, **2012**, *19(11)*, 1391-1399, DOI: 10.1016/j.chembiol.2012.07.026.
45. Fu, L., Liu, K., Ferreira, R.B., Carroll, K.S., and Yang, J., Proteome-Wide Analysis of Cysteine S-Sulfenylation Using a Benzothiazine-Based Probe. *Curr Protoc Protein Sci*, **2019**, *95(1)*, e76, DOI: 10.1002/cpps.76.
46. Yang, J., Gupta, V., Carroll, K.S., and Liebler, D.C., Site-specific mapping and quantification of protein S-sulphenylation in cells. *Nat Commun*, **2014**, *5*, 4776, DOI: 10.1038/ncomms5776.
47. Mnatsakanyan, R., Markoutsa, S., Walbrunn, K., Roos, A., Verhelst, S.H.L., and Zahedi, R.P., Proteome-wide detection of S-nitrosylation targets and motifs using bioorthogonal cleavable-linker-based enrichment and switch technique. *Nat Commun*, **2019**, *10(1)*, 2195, DOI: 10.1038/s41467-019-10182-4.
48. Lefievre, L., Chen, Y., Conner, S.J., Scott, J.L., Publicover, S.J., Ford, W.C., and Barratt, C.L., Human spermatozoa contain multiple targets for protein S-nitrosylation: an alternative mechanism of the modulation of sperm function by nitric oxide? *Proteomics*, **2007**, *7(17)*, 3066-3084, DOI: 10.1002/pmic.200700254.
49. Huang, B., Chen, S.C., and Wang, D.L., Shear flow increases S-nitrosylation of proteins in endothelial cells. *Cardiovasc Res*, **2009**, *83(3)*, 536-546, DOI: 10.1093/cvr/cvp154.
50. Majmudar, J.D., Konopko, A.M., Labby, K.J., Tom, C.T., Crellin, J.E., Prakash, A., and Martin, B.R., Harnessing Redox Cross-Reactivity To Profile Distinct Cysteine Modifications. *J Am Chem Soc*, **2016**, *138(6)*, 1852-9, DOI: 10.1021/jacs.5b06806.
51. Forrester, M.T., Thompson, J.W., Foster, M.W., Nogueira, L., Moseley, M.A., and Stamler, J.S., Proteomic analysis of S-nitrosylation and denitrosylation by resin-assisted capture. *Nat Biotechnol*, **2009**, *27(6)*, 557-9, DOI: 10.1038/nbt.1545.
52. Xu, Y., Ding, J., Wu, L.Y., and Chou, K.C., iSNO-PseAAC: predict cysteine S-nitrosylation sites in proteins by incorporating position specific amino acid propensity into pseudo amino acid composition. *PLoS One*, **2013**, *8(2)*, e55844, DOI: 10.1371/journal.pone.0055844.

53. Valek, L., Heidler, J., Scheving, R., Wittig, I., and Tegeder, I., Nitric oxide contributes to protein homeostasis by S-nitrosylations of the chaperone HSPA8 and the ubiquitin ligase UBE2D. *Redox Biol*, **2019**, *20*, 217-235, DOI: 10.1016/j.redox.2018.10.002.
54. Ben-Lulu, S., Ziv, T., Weisman-Shomer, P., and Benhar, M., Nitrosothiol-Trapping-Based Proteomic Analysis of S-Nitrosylation in Human Lung Carcinoma Cells. *PLoS One*, **2017**, *12(1)*, e0169862, DOI: 10.1371/journal.pone.0169862.
55. Liu, M., Hou, J., Huang, L., Huang, X., Heibeck, T.H., Zhao, R., Pasa-Tolic, L., Smith, R.D., Li, Y., Fu, K., Zhang, Z., Hinrichs, S.H., and Ding, S.J., Site-specific proteomics approach for study protein S-nitrosylation. *Anal Chem*, **2010**, *82(17)*, 7160-8, DOI: 10.1021/ac100569d.
56. Tan, C., Li, Y., Huang, X., Wei, M., Huang, Y., Tang, Z., Huang, H., Zhou, W., Wang, Y., and Hu, J., Extensive protein S-nitrosylation associated with human pancreatic ductal adenocarcinoma pathogenesis. *Cell Death Dis*, **2019**, *10(12)*, 914, DOI: 10.1038/s41419-019-2144-6.
57. Fu, L., Liu, K., He, J., Tian, C., Yu, X., and Yang, J., Direct Proteomic Mapping of Cysteine Persulfidation. *Antioxid Redox Signal*, **2020**, *33(15)*, 1061-1076, DOI: 10.1089/ars.2019.7777.
58. Wu, Q., Zhao, B., Weng, Y., Shan, Y., Li, X., Hu, Y., Liang, Z., Yuan, H., Zhang, L., and Zhang, Y., Site-Specific Quantification of Persulfidome by Combining an Isotope-Coded Affinity Tag with Strong Cation-Exchange-Based Fractionation. *Anal Chem*, **2019**, *91(23)*, 14860-14864, DOI: 10.1021/acs.analchem.9b04112.
